# Warfarin-related nephropathy in a patient with renal pelvic cancer 

**DOI:** 10.5414/CNCS108862

**Published:** 2017-02-03

**Authors:** Yuki Nagasako, Akiko Fujii, Satoshi Furuse, Katsunori Saito, Naobumi Mise

**Affiliations:** 1 Division of Internal Medicine, Mitsui Memorial Hospital, Tokyo, and; 2 Department of Pathology, Dokkyo Medical University Koshigaya Hospital, Saitama, Japan

**Keywords:** warfarin-related nephropathy, renal pelvic cancer, acute kidney injury, macrohematuria

## Abstract

An 83-year-old Japanese man had a history of chronic heart failure due to bradycardia-tachycardia syndrome. He was admitted to our hospital because of macrohematuria and acute kidney injury (AKI), which were detected by an urologist at an outpatient visit. He had a history of recurrent macrohematuria and transurethral resection of bladder tumors twice in the preceding 2 years. He had been on warfarin for 12 years, with a stable international normalized ratio (INR) that was usually less than 2.1. Urinalysis revealed numerous red blood cells (RBCs) and mild proteinuria without RBC casts. His serum creatinine level was elevated to 2.41 mg/dL from 0.96 mg/dL at 3 weeks before admission. INR was 1.44. Hydronephrosis was not observed. Ureteroscopy detected invasive urothelial carcinoma of the renal pelvis, and right laparoscopic nephroureterectomy was performed at 41 days after diagnosis of AKI. The background renal parenchyma displayed tubular obstruction by red blood cell casts and acute tubular injury, which were changes compatible with warfarin-related nephropathy (WRN). Warfarin was discontinued, and the serum creatinine level recovered to 1.66 mg/dL after 3 months. In the present patient with nephrosclerosis, WRN occurred at a therapeutic INR level after 12 years of uneventful warfarin therapy, and the coexisting urothelial malignancy was a unique feature.

## Introduction 

Warfarin is widely used to prevent thromboembolism in patients with various medical conditions, including atrial fibrillation and prosthetic heart valves. It has a narrow therapeutic range and interacts with many medications and foods, so warfarin therapy must be closely monitored. Hemorrhage remains the most common and serious complication of warfarin therapy, and its risk is more than two-fold higher in patients with chronic kidney disease (CKD) [1]. 

Brodsky et al. [2] first reported the occurrence of acute kidney injury (AKI) during warfarin therapy associated with obstructive tubular red blood cell (RBC) casts and termed it warfarin-related nephropathy (WRN). They speculated that glomerular hemorrhage caused by excessive anticoagulation resulted in formation of occlusive RBC casts within the renal tubules and led to tubular epithelial cell injury. Definitive diagnosis of WRN requires renal biopsy, but it is not usually performed (at least initially) because patients on warfarin therapy have an increased risk of bleeding [3]. 

In patients taking warfarin, hematuria should not automatically be attributed to impaired coagulation, since it may be a primary presenting symptom of urinary tract tumors. Thus, thorough evaluation of hematuria is important, even in patients on warfarin therapy. 

We report a patient in whom WRN occurred after 12 years of warfarin therapy with the international normalized ratio (INR) in the therapeutic range. This patient initially presented with macrohematuria due to a urinary tract tumor and later developed AKI secondary to WRN. 

## Case report 

An 83-year-old Japanese man had a history of hypertension and chronic heart failure due to bradycardia-tachycardia syndrome. He was referred to our nephrology department and admitted to hospital because of macrohematuria and AKI. He had been on warfarin since a pacemaker was implanted 12 years earlier because sinoatrial arrest was detected at that time. His INR had been stable at levels of less than 2.1, with the warfarin dose being 1 mg/day on admission. He first noticed macrohematuria 7 years before admission, which stopped spontaneously after a few days. It recurred 2 years before admission, when he underwent cystoscopy and transurethral resection of a papillary bladder tumor. Histopathological examination revealed no malignancy. However, intermittent macrohematuria persisted and worsened 7 months before admission. Transurethral resection was performed again, and the diagnosis was noninvasive papillary urothelial carcinoma (stage 0a, pTa, N0, M0). Macrohematuria still persisted and gradually became more severe, with blood clots. 10 days before admission, cystoscopy revealed a jet of bloody urine from the right ureteral orifice without apparent recurrence of the bladder tumor. At outpatient follow-up, acute deterioration of renal function was detected, and he was admitted to hospital. 

On admission, physical examination revealed right costovertebral angle tenderness and mild pitting edema of the bilateral lower extremities. Urinalysis showed numerous RBCs without casts. The urine protein to creatinine ratio was 0.17. His sCr was 2.41 mg/dL, although it had been 0.96 mg/dL (eGFR; 57.1 mL/min/1.73m^2^) at 3 weeks prior to admission. In addition, hemoglobin was 9.2 g/dL, C-reactive protein was 0.9 mg/dL, and INR was 1.44. Contrast-enhanced computed tomography (CT) demonstrated a high-density area extending from the right kidney to the ureter and deformity of the inferior pole of the right kidney, which had not changed for 2 years. No hydronephrosis was detected. 

Macroscopic hematuria persisted after discontinuation of anticoagulant therapy. Ureteroscopy identified thick blood clots in the upper to middle part of the ureter, and intravenous pyelography (IVP) detected a right renal papillary tumor. Biopsy and washing cytology of the right renal pelvis revealed urothelial carcinoma, so right laparoscopic nephroureterectomy was performed at 41 days after the diagnosis of AKI. 

The resected specimen measured 33×11×2 cm and included the kidney (12×11×2 cm). Macroscopic examination showed a 2-cm, poorly-demarcated, ash-colored papillary tumor in the right renal pelvis, while microscopy revealed urothelial carcinoma invading the renal parenchyma beyond the muscular layer (stage III, pT3, N0, M0). 

**Figure 1.**
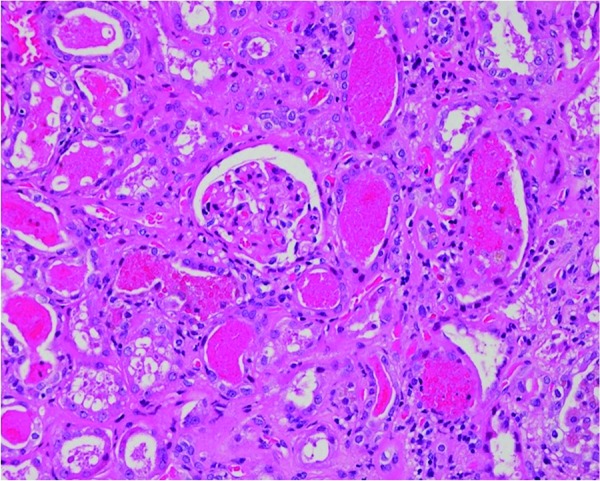
Renal parenchyma, H & E, ×200. The renal tubules are filled with numerous erythrocytes. Acute tubular injury is evident, with loss of the brush border and nuclear enlargement. H & E, ×200.

**Figure 2.**
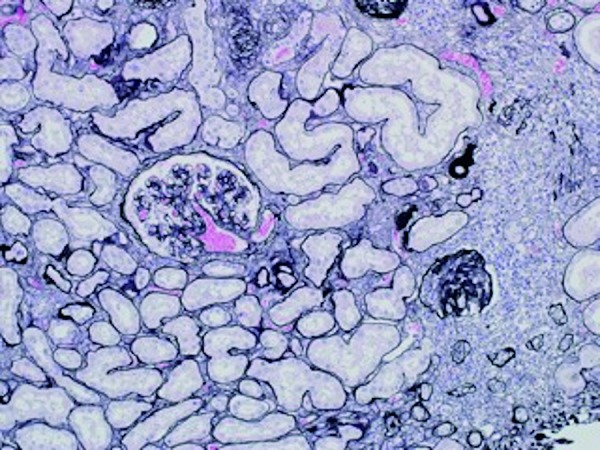
Glomeruli show ischemic changes, with corrugation of the glomerular basement membrane and global sclerosis, accompanied by focal interstitial fibrosis and tubular atrophy. Jones methenamine silver stain (PAM), ×200.

In the kidney, the tubules were filled with RBCs and some cell debris. The tubular epithelial lining was flattened due to distension by RBC casts. Some of the tubules showed acute injury with loss of the brush border and regenerative changes characterized by nuclear enlargement (Figure 1). There was mild interstitial fibrosis with mononuclear cell infiltration. Focal glomerular ischemic changes were noted, with corrugation of the glomerular basement membrane and global sclerosis (Figure 2). Interlobular arteries showed moderate arteriosclerosis, but there was no hyalinosis. Frozen sections were negative for staining with IgG, IgA, IgM, C3, C4, κ-light chain, and λ-light chain. Electron microscopy revealed a slight increase of the mesangial matrix, but no electron-dense deposits were identified. 

Initially, sCr rose to 2.96 mg/dL, but it recovered to 1.66 mg/dL after 3 months. His macrohematuria resolved 2 days after nephroureterectomy, and there was no recurrence of hematuria 8 months after surgery. Warfarin therapy was not resumed. 

## Discussion 

The present case has several unique features. First, the patient presented with macrohematuria caused by recurrent urinary tract tumors and later developed WRN. Second, WRN occurred while the INR was in the therapeutic range. Third, there were only slight underlying glomerular abnormalities. Fourth, WRN occurred after 12 years of warfarin treatment. 

Patients taking anticoagulants have a similar risk of major genitourinary diseases as patients not on anticoagulant therapy, and bleeding may be the first sign of these disorders. In a prospective study of patients with either microscopic or macroscopic hematuria, 39.5% had identifiable pathology, including bladder cancer (11.9%), renal cancer (0.6%), and urothelial cancer (0.1%) [4]. Gross or microscopic hematuria was the principal sign of renal pelvic or ureteral cancer and was present in more than 75% of patients at diagnosis [5]. Therefore, hematuria in patients on warfarin therapy should not be attributed to hypocoagulation, and thorough evaluation is important. 

In this patent, acute aggravation of macrohematuria was observed at the onset of AKI. The main cause of the hematuria seemed to be renal pelvic cancer because no other probable sources of bleeding were detected, and hematuria improved drastically after nephroureterectomy. However, WRN might have contributed to both hematuria and AKI. In the resected kidney, acute tubular injury was observed with occlusion of tubules by RBC casts, while the glomeruli showed mild ischemic changes. Immunofluorescence studies were negative, and no electron-dense deposits were found by electron microscopy. These findings are compatible with those previously reported for WRN on a background of chronic kidney disease caused by 20 years of hypertension. No glomerular hemorrhage was seen, probably because glomerular bleeding had already ceased at the time of nephrectomy, which was performed more than 1 month after diagnosis of AKI and discontinuation of warfarin. 

A definite diagnosis of WRN can only be made by histopathological examination, but WRN is usually diagnosed clinically, presumably due to the high risk of bleeding if renal biopsy is performed. Thus, the actual incidence of WRN is difficult to determine, and most studies have employed presumptive diagnosis, based on elevation of serum creatinine within several days after detection of an abnormally high INR. Hematuria is common, but microscopic hematuria is more frequent than macrohematuria [2, 6, 7]. Hematuria usually occurs within 8 weeks of starting warfarin [6, 7, 8], but the interval between elevation of the INR and the onset of AKI is unclear. The average INR of patients with WRN has been reported to be in the range of low to mid 4s [6], but the correlation between INR and the severity of AKI has not been clarified [2]. In the present patient, the INR was 1.66 upon diagnosis of AKI. Brodsky et al. [2] reported a case of WRN with an INR of 2.0, which suggests that this disorder may sometimes occur when the INR is in the therapeutic range. It is also possible that our patient temporarily developed excessive anticoagulation at the onset of AKI after which the INR returned to the therapeutic range by the time of diagnosis. In our patient, WRN occurred after 12 years of warfarin treatment. Because he had hypertension, nephrosclerosis may have developed gradually and structural changes of the glomeruli could have enhanced vulnerability to WRN. Indeed, Brodsky et al. [2] suggested that the combination of even mild glomerular disease and warfarin-induced coagulopathy could result in glomerular hematuria and significant accumulation of RBCs within the nephrons [2]. 

After discontinuation of warfarin and nephroureterectomy, renal function recovered almost fully, considering the fact that he only had one kidney. Taken together with the persistence of microscopic hematuria for a long period before nephroureterectomy, similar subclinical pathophysiology might have occurred repeatedly. WRN may lead to irreversible kidney injury and CKD and has been associated with an increased risk of mortality in some patients [6, 10]. In previous studies, the risk factors for WRN were found to be the age, diabetes, hypertension, cardiovascular disease, CKD, and low basal serum albumin level [2, 10]. Our patient was elderly, hypertensive, and had CKD with benign nephrosclerosis, so he had three of these risk factors. 

To our knowledge, this is the first reported case with a pathologic diagnosis of renal pelvic cancer complicated by WRN. This case is also unique in that WRN occurred after 12 years of warfarin therapy while the INR was in the therapeutic range. Careful evaluation of hematuria in patients using warfarin is important for the diagnosis of WRN. 

## Conflict of interest 

The authors declare no conflict of interest.
